# Thorough clinical child psychiatric diagnostic evaluation and validation of the Autism- Tics, ADHD and other comorbidities inventory (A-TAC) in a population-based sample of 9-year-olds

**DOI:** 10.1186/s12888-025-07475-y

**Published:** 2025-10-02

**Authors:** Linda Halldner, Sophia Eberhard, Paul Lichtenstein, Peik Gustafsson, Christopher Gillberg, Mats Johnson, Eva Billstedt, Jakob Täljemark, Maria Råstam, Sebastian Lundström

**Affiliations:** 1 Department of Clinical Sciences, Child and Adolescent Psychiatry, Umeå University, ZC23, by 28, NUS, Umeå, SE-901 87 Sweden; 2 Department of Medical Epidemiology and Biostatistics, Karolinska Institutet, Stockholm, Sweden; 3 Department of Clinical Sciences, Child and Adolescent Psychiatry, Lund University, Lund, Sweden; 4 Department of Psychiatry and Neurochemistry, Gillberg Neuropsychiatry Center, University of Gothenburg, Gothenburg, Sweden

**Keywords:** Children, Neurodevelopmental disorders, Psychiatric diagnoses, Screening validation, Clinical evaluation

## Abstract

**Background:**

The Autism- Tics, ADHD and other Comorbidities inventory (A-TAC) has been validated in epidemiological data. However, validation against clinical diagnostic assessments in a population-based sample has been lacking, limiting the implications for clinical practice, clinical research and public health decisions.

**Methods:**

Study participants were recruited from the longitudinal Child and Adolescent Twin Study in Sweden (CATSS) inviting parents to all twins in Sweden. We investigated the psychometric properties of the A-TAC in 263 children, where one or both twins screened positive for neuropsychiatric problems, as well as control pairs, where both twins were screen negative. Study participants underwent thorough clinical examination within one year of the A-TAC interview. The psychometric properties of the A-TAC were then investigated. We also mapped the extent of comorbidity of neurodevelopmental disorders.

**Results:**

Using the A-TAC as screening for neurodevelopmental disorders we could discriminate two groups of children with clearly different occurrences of clinical diagnoses. The predictive screening properties of the A-TAC were good for most of the neurodevelopmental disorders (AUC ranging from 0.806 to 0.958), with exception for developmental coordination disorder (AUC = 0.616). More than 40% of children fulfilling diagnostic criteria for a neurodevelopmental disorder, also fulfilled diagnostic criteria for at least one other neurodevelopmental disorder.

**Conclusion:**

This study confirms the utility of the A-TAC interview as a screening tool for neuropsychiatric disorders in a non-clinical sample. It also supports the necessity to maintain a broad diagnostic approach in clinical child psychiatric investigations for meaningful understanding of the child’s problems. Although, A-TAC can be informative on neurodevelopmental problems in both clinical and population-based samples, it cannot replace a clinical neurodevelopmental investigation or be used to delimit individual access to specialized care.

**Supplementary Information:**

The online version contains supplementary material available at 10.1186/s12888-025-07475-y.

## Background

The demand for clinical investigations of neurodevelopmental disorders (NDD) has escalated the latest decades. Meanwhile, increased mental health issues in children and adolescents overall have been reported [1–3], leading to a considerable strain on the Child and Adolescent Mental Health Services (CAMHS). Taken together CAMHS need means to efficiently direct existing resources. Validated screening tools are suitable for this purpose and are crucial to evaluate and further plan individual mental health care, as well as mental health care at an organizational level. For example, targeting populations suitable for early interventions can relieve excess referrals to specialized care, leaving proper health care resources for individuals with clear need for specialized care. Also, screening can help identify problem areas subject for further clinical investigation on an individual level, avoiding focus on one single highlighted problem area.

The true prevalence of different neurodevelopmental and psychiatric diagnoses in the pediatric population is often debated [2, 4, 5]. Estimations of the presence of comorbidity panoramas are especially challenging [6, 7] and most prevalence studies or instruments focus on single disorders, not taking into account the vast coexistence [8].Taken together, validated screening tools suitable for both clinical and nonclinical samples and covering several problem areas, are needed for this purpose.

The Autism- Tics, ADHD and other Comorbidities inventory (A-TAC) is a comprehensive assessment tool that encompasses all major clinical diagnostic criteria in child and adolescent psychiatry, beginning with ASD and systematically addressing other neurodevelopmental disorders known to overlap with ASD (including ADHD, TDs, DCD, and LDs), as well as most other relevant domains of child and adolescent psychopathology. The questions were formulated to capture DSM symptom definitions and diagnostic criteria, as well as well-known clinical characteristics by a team of experts from Gillberg Neuropsychiatry Centre at the University of Gothenburg. It has been validated cross-sectionally [9–12] and longitudinally [13]. It has also been validated against diagnoses in the national patient register [14], and has shown excellent psychometric properties for ADHD, autism, and other NDDs in epidemiological settings and in clinical practice. However, a validation by in-person, clinical diagnostic assessments in a large population-based sample in close conjunction with the screening procedure, remains. While registry-based validations provide broad epidemiological insights, in-person clinical assessments allow for more nuanced diagnostic precision, reducing potential biases from administrative coding.

In this study we aimed to assess sensitivity, specificity, positive predictive value, negative predictive value, diagnostic odds ratio as well as calculating area under curve (AUC) by using receiver operating characteristic curves, of the A-TAC against gold-standard clinical evaluations in a sample of 9–10-year-olds. This age group was chosen to perform clinical assessments within one year of the A-TAC screening interviews in the CATSS-study (see below). This age group further constitutes an age span when NDD problems become more evident due to increased academic and social demands, but also that the majority of major child psychiatric disorders are typically established, whereas difficulties associated with puberty have generally not yet manifested. A secondary aim was to investigate the patterns and extent of comorbidity of NDDs.

## Methods

### Population

Study participants were recruited from the longitudinal Child and Adolescent Twin Study in Sweden (CATSS) which has been described in detail elsewhere [15]. The CATSS study emanates from the Swedish twin registry. CATSS is a large national attempt to study neurodevelopmental and neuropsychiatric problems in children with both epidemiological and twin techniques. Briefly, parents to all twins in Sweden (roughly 1400 twin pairs annually) born July 1992 onwards were contacted for participation in CATSS. Consenting parents were invited to participate in a telephone interview in connection with the twins ninth birthday. CATSS has a response rate of 70–80% with small differences between responders and non-responders with regards to neuropsychiatric problems when compared to the National Patient Register [15]. The CATSS interview contains the Autism – Tics, AD/HD, and other Comorbidities Inventory (A-TAC, see below) which is used to screen for neuropsychiatric problems. The CATSS-clinical study was designed to investigate a subpopulation clinically, to strengthen the validity of any data from the CATSS population, and to validate the A-TAC on a population-based cohort. Previous results imply that the twin population in most areas is similar to singlet populations [16–18].

In the present project (CATSS-clinical), all twin pairs within the CATSS residing in the regions of Stockholm, Västra Götaland and Skåne, and where one or both twins screened positive for an NDD, CD, ODD, OCD, and/or eating problem in the telephone.

interviews, were invited to participate in a clinical neurodevelopmental assessment at the regional site within a year of their screening interview. We also invited 5% control pairs, i.e. twin pairs from CATSS from the same regions, where both twins were screen negative. There were no exclusion criteria for the CATSS-clinical study, but the assessment had to be within a year after the telephone/A-TAC-interview. During the time period January 2011 – October 2014 in total 346 twin pairs were eligible for the present study. Of these 346, 90 pairs (27%) could not be reached, 112 pairs (32%) declined participation, 12 pairs (3%) accepted but discontinued participation before the clinical examination. Finally, 132 pairs (38%) attended the clinical examination. In one case only one of the twins attended the clinical examination resulting in a total of 263 clinically examined 9–10-year-old children. Of the children participating in the assessment 46% were screen-positive from the A-TAC interview, the remaining part were controls or screen-negative co-twins. The clinical investigators were blinded to the case-ness. Part of the CATSS-clinical population and the instruments have been described in previous publications [19].

### Screening (CATSS)

As previously mentioned, the A-TAC was used to identify individuals with neuropsychiatric problems. The A-TAC consists of 96 questions that are answered from a life-time perspective. Questions are answered “Yes” (scored as 1), “Yes, to some extent” (scored as 0.5) and “No” (scored as 0) and taps in to virtually all common child and adolescent psychiatric problem constellations.

The inventory shows excellent psychometric properties for ADHD, autism, and other NDDs in epidemiological settings and in clinical practice [9–14]. In addition, it has excellent inter- and intra-rater reliability [13]. The exact algorithms for inclusion can be found in the endnotes by Anckarsäter et al. [15].

### Clinical examination (CATSS-clinical)

Consenting families were invited to a clinical examination where two experienced clinicians (psychologist, social worker, or resident in child and adolescent psychiatry), blind for the A-TAC data, conducted the clinical examinations. The clinicians had received special training (e.g. certified K-SADS-training, or corresponding individualized training) in the use of the instruments before the study. Most of the participating clinicians had additional experience in using the instruments in a previous similar study. The results and the complete collected materials were validated by a senior child psychiatrist and a decision of a clinical diagnosis was made in consensus with the assessing clinician. In the rare case of disagreement, the senior child psychiatrist made the final decision on the diagnosis. The best diagnoses describing the child’s problems were chosen based on the DSM-IV classification, except that we allowed simultaneous ASD and ADHD diagnoses. In a first step the parent filled out questionnaires regarding ADHD (the Swanson, Nolan, and Pelham ADHD Rating Scale-version IV, SNAP-IV [20]) and autism (the Autism Spectrum Screening Questionnaire, ASSQ [21]) respectively, including permission for requisition of relevant medical records. Together with an accompanying parent, one of the twins was interviewed with the Kiddie-SADS-Present and Lifetime Version, covering most psychiatric diagnoses in children, the Asperger Syndrome (and high-functioning autism) Diagnostic Interview (ASDI), and the sensory deviations section from the Diagnostic Interview for Social and Communication Disorders schedule (DISCO), a separate semi structured interview on developmental milestones (on e.g. language and motor functions), and the child underwent a brief clinical neurological investigation, while the other twin was assessed with the WISC-IV (Wechsler intelligence scale for children). The procedure was then reversed, so that both twins underwent WISC-IV assessment as well as were subjects for the interview.

### Instruments in CATSS-clinical

In CATSS-clinical the Kiddie-SADS-Present and Lifetime Version 1.0, (K-SADS) [22] was used. It is a semi-structured child psychiatric diagnostic interview with child and parent. The version used in this study captures both present and former diagnoses. The K-SADS contains of questions coupled to DSM-IV criteria first in screening fashion, and if screen-positive, then an in-depth-interview regarding the specific disorder(s). K-SADS is widely used in clinical as well as research practices. For the present study K-SADS was chosen to collect a broader clinical picture of present symptoms enabling proper differential diagnosis considerations. Furthermore, the section covering sensory deviations from the Diagnostic Interview for Social and Communication Disorders schedule (DISCO) [23, 24] was included, as sensory deviations were not included in the DSM-IV diagnostic criteria. In addition, the Asperger Syndrome (and high-functioning autism) Diagnostic Interview (ASDI) [25], a brief semi-structured parent interview, which consists of 20 questions on symptoms of autism spectrum disorder, due to the clinical experience of poor qualities of the K-SADS to delineate autism spectrum disorders. Further the ADHD & Autismspektrumtillstånd – Observationshjälpmedel (A&O), which is a structured clinician-rated psychiatric observation tool [26] was included to obtain an objective measure of psychiatric status. Finally, the WISC-IV provided standard scores with a mean of 100 and a standard deviation of 15. The Swedish version is based on British norms, which have been tried out and validated against Swedish populations [27]. WISC is an internationally well validated, and widespread means of objectively assessing intelligence in children.

### Statistical analyses

Validation of the A-TAC interview against clinical diagnosis were made using receiver operating characteristics curves (ROC) to calculate the area under curve (AUC) and thus sensitivity, and specificity, respectively. In addition, and to allow for comparison with other instruments, diagnostic odds ratios, positive predictive values, negative predictive values, as well as accuracy were calculated using the previously defined cut-offs for different A-TAC screening scores [10, 14]. For ADHD, autism spectrum disorder (ASD), intellectual disability (ID), and developmental coordination disorder (DCD), two predefined cut-offs -one higher and one lower- were available. As the A-TAC interview is asking for lifetime symptoms the validation was made against the presence of ever having fulfilled the clinical diagnosis (either a present or a former diagnosis). Co-morbidity was defined as the presence of more than one neurodevelopmental or psychiatric diagnosis and thus analyzed as a binary variable.

The software IBM SPSS Statistics version 28.0.1.1 [14]. receiver operating characteristics curves tool was used for AUC calculations, and assessment of sensitivity and specificity. Calculation tools at www.medcalc.org were used for calculation of diagnostic odds ratios, positive predictive values, and negative predictive values.

## Results

### Clinical diagnoses and comorbidity

The frequencies of the present clinical diagnoses are shown in Table 1. The most prevalent diagnosis in the clinically investigated sample was ADHD (17.5%), followed by tic disorders (10.3%). As expected, when investigating a sample of which a large proportion was screen-positive for neuropsychiatric disorders, the prevalence of autism spectrum disorders was relatively high, > 5%. Of the 120 children classified as controls from the A-TAC interview, 101 were found not to fulfill diagnostic criteria for any NDD; here defined as autism spectrum disorder, ADHD, tic disorder, developmental coordination disorder, intellectual disability, oppositional defiant disorder, conduct disorder, obsessive-compulsive disorder, or early onset restrictive eating disorder.

**Table 1:** Current clinical diagnoses in the 263 individuals who underwent the clinical investigation. Current but subsyndromal refers to individuals with former full diagnosis but in current partial remission. NOS = not otherwise specified, OCD = obsessive-compulsive disorder, DCD = developmental coordination disorder, GAD = generalized anxiety disorder, PTSD = posttraumatic stress disorder, PDD = pervasive developmental disorder

diagnosis	current		current but subsyndromal		
	*n*	%	*n*	%	
Substanse abuse/dependence	0	0	0	0	
Eating disorders	1	0.4	0	0	
Bipolar/psychotic disorders	0	0	0	0	
Tics/Tourette´s disorders	27	10.3	5	1.9	
Transient tic disorder	1	0.4	1	0.4	
Chronic motor or vocal tic disorder	15	5.7	1	0.4	
Tourette´s disorder	8	3.0	3	1.1	
Tic disorder NOS	3	1.1	0	0	
OCD	2	0.8	0	0	
DCD	3	1.1	0	0	
Selective mutism	1	0.4	0	0	
Specific phobia	13	4.9	3	1.1	
Social phobia	1	0.4	1	0.4	
Separation anxiety disorder	6	2.3	6	2.3	
Panic disorder	3	1.1	1	0.4	
Agoraphobia	2	0.8	0	0	
GAD	4	1.5	0	0	
Anxiety disorder NOS	4	1.5	2	0.8	
PTSD	1	0.4	2	0.8	
Adjustment disorders	2	0.8	1	0.4	
Major depressive disorder	2	0.8	0	0	
Depressive disorder NOS	0	0	3	1.1	
Dysthymic disorder	0	0	2	0.8	
Asperger Syndrome	3	1.1	0	0	
PDD-NOS	13	4.9	0	0	
Autistic disorder	1	0.4			
ADHD	46	17.5	0	0	
combined subtype	16	6.1	3	1.1	
hyperactive-impulsive subtype	9	3.4	0	0	
inattentive subtype	23	8.7	1	0.4	
ADHD NOS	9	3.4	0	0	
Oppositional Defiant Disorder	14	5.3	1	0.4	
Conduct disorder	1	0.4	0	0	
Disruptive Behavior Disorder NOS	5	1.9	0	0	
Enuresis	13	4.9	8	3.0	
Encopresis	8	3.0	2	0.8	
Sleep disorders	5	1.9	0	0	
Dyslexia	2	0.8	0	0	
Communication disorders	5	1.9	0	0	
Intellectual Disability	7	2.7	0	0	

Of the total 92 children with confirmed NDD (73 from the screen positive group and 19 from the negative screen group), > 40% (n **=** 37) met criteria for more than one NDD. In addition, a substantial presence of comorbidity with anxiety disorders (including separation anxiety, panic disorder, specific phobia, agoraphobia, generalized anxiety disorder, and anxiety NOS) and trauma related diagnoses (including PTSD, acute stress disorder, adjustment disorders), was found, see Table 2.

**Table 2:** Showing the number of children with number of clinical neurodevelopmental diagnoses (NDDs, including autism spectrum diagnoses, ADHD, tic disorders, developmental coordination disorder, intellectual disability, ODD, CD, OCD, and eating disorders) and comorbidity with anxiety diagnoses (including separation anxiety, panic disorder, specific phobia, agoraphobia, GAD and anxiety NOS) and trauma related diagnoses (including PTSD, acute stress disorder, adjustment disorders). Cases = children screen-positive from the A-TAC interview, Controls = children screen-negative from the A-TAC interview

A-TAC status	Nr diagnoses	0	1	2	3	4	5	6
cases	NDDs	66	40	21	10	1	1	0
	NDDs + anxiety	56	40	26	9	7	0	1
	NDDs + anxiety + trauma	55	40	24	12	7	0	1
controls	NDDs	101	15	3	1	0	0	0
	NDDs + anxiety	87	24	7	2	0	0	0
	NDDs + anxiety + trauma	84	27	7	2	0	0	0
all	NDDs	167	55	24	11	1	1	0
	NDDs + anxiety	143	64	33	11	7	0	1
	NDDs + anxiety + trauma	139	67	31	14	7	0	1

### Screening and psychometric properties of the A-TAC interview (see Table 3, suppl Fig. 1)

Of the 139 investigated children defined as cases from the A-TAC interview, 73 were found to fulfil criteria for at least one NDD (see Table 2). Thus, of the screen-positive participants 52.5%, and only 15.8% of the screen-negative participants, fulfilled criteria for at least one NDD diagnosis after clinical investigation. This can be interpreted as A-TAC being a suitable tool for distinguishing between two different populations.

The predictive screening properties of the A-TAC were good for most of the NDDs here specifically investigated. AUC values ranged from 0.616 (poor; for DCD) to 0.923 (outstanding; for OCD). As only one study participant was assessed to fulfill for each CD and ED respectively, the psychometric results on validity for these A-TAC items have to be interpreted with caution. Of note was that the two cut-off values validated for ADHD and ASD respectively differed primarily on the PPV, and less so on the NPV (see Table 3), suggesting the A-TAC to be a relatively good screening-tool for ruling out these diagnoses also at the lower cut-offs.

**Table 3:** Validation of ATAC screening interview against clinical diagnoses. (AUC = area under curve with 95% confidence intervals within brackets), SENS = sensitivity, SPEC = specificity, PPV = positive predictive value, NPV = negative predictive value, DOR = diagnostic odds ratio)

diagnosis	AUC	A-TAC cut-off	SENS	SPEC	PPV	NPV	Accuracy	DOR
ADHD	0.806	6	0.661	0.772	45.88	88.64	74.71	6.6
	(0.747;0.866)	12.5	0.203	0.990	85.71	80.97	81.23	25.53
ASD	0.866	4.5	0.500	0.934	38.46	95.74	90.04	14.06
	(0.792;0.939)	8.5	0.250	0.988	62.50	94.07	93.10	26.44
tics	0.828	1.5	0.439	0.945	60.00	90.00	86.54	13.50
	(0.747;0.910)							
OCD	0.923	1	0.333	0.926	5.00	99.17	91.95	6.28
	(881;0.966)							
ED	0.958	1	1.00	0.924	4.17	100.00	91.19	1.04
	(0.907;1.00)							
ODD	0.889	3	0.923	0.822	21.43	99.51	82.69	55.36
	(0.806;0.971)							
ID	0.914	1	1.00	0.780	11.11	100	78.54	1.12
	(0.865;0.963)	3	0.143	0.972	12.50	97.63	95.02	5.88
DCD	0.616	0.5	0.333	0.860	2.70	99.11	85.44	3.08
	(0.239;0.994)	1	0.333	0.977	14.29	99.21	96.93	21.00
CD	0.903	2	0	0.965	-	-	-	-
	(0.846;0.960)							

## Discussion

This study aimed at investigating the validity of the A-TAC instrument in a large population-based sample of clinically investigated 9-year-olds. The A-TAC proved to be a good to outstanding screening instrument for capturing the different neurodevelopmental diagnoses specifically investigated, except for screening for developmental coordination disorder (DCD). The latter is somewhat surprising as A-TAC was an excellent screening tool for detecting OCD. Both DCD symptoms and OCD symptoms (mostly) entail behaviors that should be easy to detect for the surrounding. One could consider that it is hard for parents to tell if observed “clumsiness” is due to inattention or to poor motor coordination. Also, the awareness of ADHD is commonly spread in Sweden, whereas DCD is rather unknown in the society, and is also poorly investigated in the clinical context. It may also be that the A-TAC interview simply is not formulated in a good way to catch coordination problems. However, the results for OCD screening were also based on few individuals fulfilling OCD criteria in the clinical assessment, and the results may thus be interpreted with some caution.

The predictive value of the A-TAC screening in this study with clinical investigations within a year after the A-TAC interview, was overall slightly better than the predictive value when screening was made 3 years before a clinical investigation [13]. For example, the positive predictive value (PPV) for ADHD using the higher cut-off was 63% in the follow-up at 15 years, compared to a PPV of 86% for the same cut-off in the present study. The corresponding PPVs for the higher cut-off for ASD were 60% and 62.5%, respectively. The differences probably reflect the overlap and time changes in the clinical symptom panorama related to neurodevelopmental problems [28]. The usage of A-TAC in the child and adolescent psychiatry warrant some considerations. In general, the specificity with the cut-offs we used were good, suggesting that the A-TAC tool is suitable to estimate the presence of clinical diagnostic cases also when used in population-based samples. However, the sensitivity of the pre-defined cut-offs was lower, which may limit its use for selection for clinical neurodevelopmental investigations. This may further highlight that clinical investigations with too targeted diagnostic questions are not useful for the individual patient. That is, if clinical interviews, in the interest of time efficiency, are too slim, the patient´s symptoms or functional deficits risk to be misinterpreted or not acknowledged at all. Despite this, from our results, and particularly the calculated diagnostic odds ratios reflecting the discriminative properties, it seems that the higher cut-offs would probably be more useful in a clinical context. While the lower cut-offs could be more useful in screening settings if the purpose is to identify individuals that are most likely *not* cases for specialized psychiatric care. In the present study using the A-TAC screening tool, we could define two groups of children (one screen-positive and one screen-negative) with clearly different prevalence of neuropsychiatric problems fulfilling diagnostic criteria. How these reflect future diagnostic panoramas or future social functioning and what predicts different life trajectories remains to be investigated in future studies.

Our results showed a widespread comorbidity between different NDDs but also comorbidity with other psychiatric problems. This reflects the known shared genetics underpinnings of different NDDs [29] and the vast symptomatic overlap [30]. Also, there are downstream biological morphological measures reflecting overlapping symptom profiles between different NDDs [31, 32]. Emerging evidence also highlights overlapping neurobiological underpinnings between NDDs and affective disorders [33]. Although this may somewhat question the delineations of psychiatric diagnoses, it underscores the importance of discriminating clinical cases with need for specialized care against milder symptoms frequent in the population. As such, the A-TAC could serve as one useful tool.

Of note, the prevalence of DCD in our sample was low (1.1%). According to Biotteau et al. [34] the prevalence of DCD is 1.8–8.8% depending on the diagnostic criteria used. In children with e.g. ADHD the prevalence has been estimated as substantially higher [8]. DCD is characterized by slow, clumsy, or inaccurate motor learning and motor performances. It might be that, since abandoning the concept of “deficits in attention, motor control and perception”, DAMP [35], these problems and symptoms are probably not acknowledged in neurodevelopmental investigations. Possibly, when specifically asked for, these symptoms are interpreted as signs of other problems such as e.g. inattention. In the present study brief coordination testing (e.g. diadochokinetic testing) was part of the clinical investigation. This indicates that this kind of test does not easily detect deficits in motor coordination that are not part of a specific neurological disorder. Deficits in rhythm and timing seems to be a common feature in most neurodevelopmental disorders [36] and was recently shown to be predictive of ADHD diagnosis [37]. Further, social interaction and motor problems in particular predicted later bully victimization [38]. Finally, the low DCD prevalence may have inflated false negatives, suggesting A-TAC’s DCD sensitivity could improve in samples enriched for motor deficits. Thus, DCD symptoms may be an area of interest for future studies,

### Limitations

Firstly, many of the eligible children were not reached or declined participation in the present study. Secondly, as the investigated population contained many children selected for neuropsychiatric disorders, our prevalence figures cannot be generalized to the overall population. Third, the population was recruited from the three most inhabited regions in Sweden, with an underrepresentation of more sparsely populated areas. We cannot rule out that the results could be less generalizable for populations in rural areas. However, results from the present study highlights the A-TAC as a valid instrument for prevalence estimates in non-clinical samples of children. A fourth limitation of the present study is the low number of children fulfilling clinical diagnostic criteria for conduct disorder, eating disorders and intellectual disability, which limited the possibility to estimate the predictive validity of the A-TAC for these disorders in particular. None of these disorders are however expected to be more prevalent in a population of 9–10-year-old children. Thus, a larger sample may be needed to investigate the psychometric properties for these A-TAC domains. In a previous A-TAC validation against previous diagnoses in the national patient register in a larger sample the psychometric properties of the A-TAC the previous validity was acceptable (AUC 0.72) for ED, and excellent (AUC 0.90) for CD [14].

## Conclusion

This study confirms the utility of the A-TAC interview as a screening tool for neuropsychiatric disorders in a non-clinical sample. Although, A-TAC can be informative on neurodevelopmental problems in both clinical and population-based samples, it cannot replace a clinical neurodevelopmental investigation or be used to delimit individual access to specialized care. In line with previous extensive literature the rate of comorbid neurodevelopmental as well as other child psychiatric disorders and trauma-related disorder was high. For this reason, our data adds to the fact that clinical child psychiatric investigations should maintain a broad diagnostic approach, and that diagnostically directed clinical child psychiatric investigations lack foundation.

## Supplementary Information

Supplementary Material 1.

## Data Availability

According to the ethical permit for the study, the data that support the findings of this study are not publicly available. According to (anonymized information) regulations, the data are classified as sensitive personal data. The data are accessible upon specific request from the corresponding author.

## Abbreviations

A&O: ADHD & Autismspektrumtillstånd – Observationshjälpmedel
ADHD: Attention deficit hyperactivity disorder
ASD: Autism spectrum disorder
ASDI: Asperger Syndrome (and high-functioning autism) Diagnostic Interview
AUC: Area under curve
ASSQ: Autism Spectrum Screening Questionnaire
A-TAC: Autism- Tics, ADHD and other Comorbidities inventory
CATSS: Child and Adolescent Twin Study in Sweden
CAMHS: Child and Adolescent Mental Health Services
DAMP: Deficits in attention, motor control and perception
DCD: Developmental coordination disorder
DISCO: Diagnostic Interview for Social and Communication Disorders schedule
ID: Intellectual disability
K-SADS: Kiddie-SADS-Present and Lifetime Version 1.0
NDD: Neurodevelopmental disorders
PPV: Positive predictive value
ROC: Receiver operating characteristics curves
SNAP-IV: Swanson, Nolan, and Pelham ADHD Rating Scale-version IV
WISC-IV: Wechsler intelligence scale for children
