# Supplementary material for: Thorough clinical child psychiatric diagnostic evaluation and validation of the Autism- Tics, ADHD and other comorbidities inventory (A-TAC) in a population-based sample of 9-year-olds
Source: BMC Psychiatry. 2025 Oct 2;25:918. doi: 10.1186/s12888-025-07475-y (PMC12490148; doi:10.1186/s12888-025-07475-y)
Supplement: Supplementary file 1 — Supplementary Material 1. [file 12888_2025_7475_MOESM1_ESM.docx]

**a)**


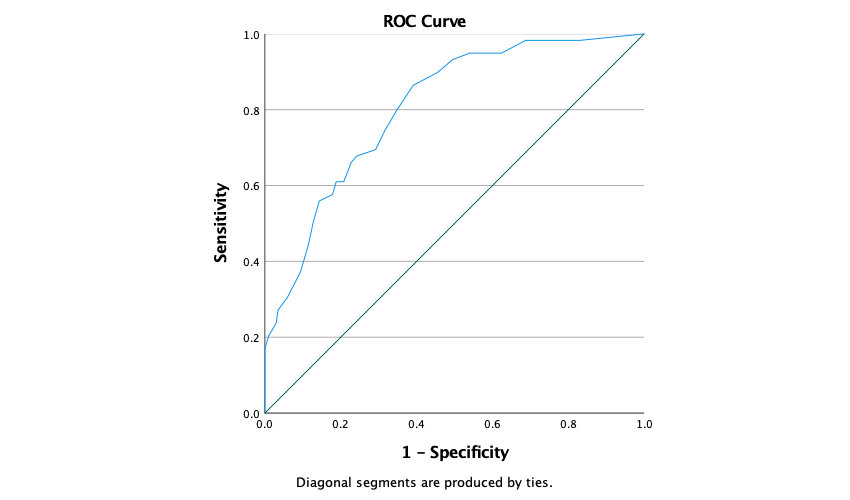


**b)**


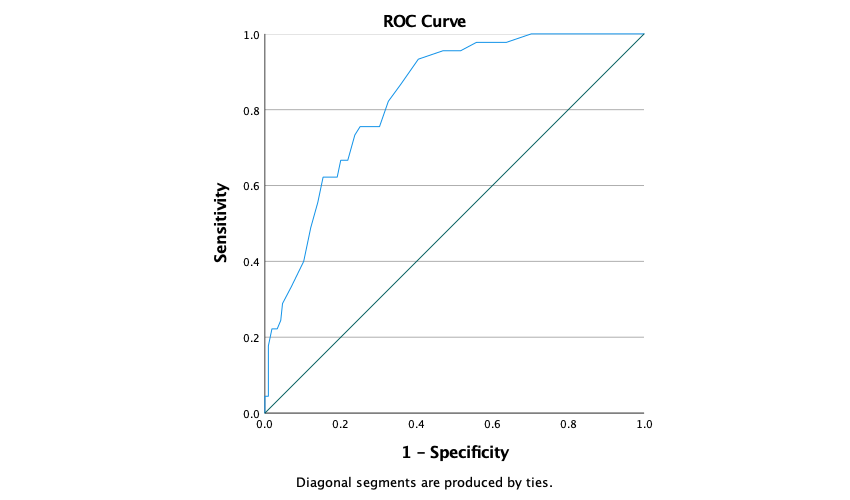


**Supplemental Figure 1** Receiver Operating Characteristic (ROC) curves demonstrating the predictive ability of the Autism- Tics, ADHD, and other Comorbidities inventory (A-TAC) for the detection of a) ADHD diagnosis, and b) autism spectrum disorder (ASD).
